# Novel Biobased Textile Fiber from Colombian Agro-Industrial Waste Fiber

**DOI:** 10.3390/molecules23102640

**Published:** 2018-10-15

**Authors:** Maria Camila Amaya Vergara, Melissa Paola Cortés Gómez, Maria Clara Restrepo Restrepo, Jorge Manrique Henao, Miguel Angel Pereira Soto, Piedad Felisinda Gañán Rojo, Cristina Isabel Castro Herazo, Robin Zuluaga Gallego

**Affiliations:** 1 School of Engineering, Universidad Pontificia Bolivariana, Circular 1 # 70-01, Medellín 56006, Colombia; mcavam@gmail.com (M.C.A.V.); melic08@hotmail.com (M.P.C.G.); jorge.manrique@upb.edu.co (J.M.H.); piedad.ganan@upb.edu.co (P.F.G.R.); robin.zuluaga@upb.edu.co (R.Z.G.); 2 School of Architecture and Design, Universidad Pontificia Bolivariana, Circular 1 # 70-01, Medellín 56006, Colombia; mariaclararestreporestrepo@gmail.com; 3 Chemical Engineering Department, Universidad de Concepción, Edificio Gustavo Pizarro Castro, 2do Piso, Barrio Universitario, Concepción 4030000, Chile; miguelpereira@udec.cl

**Keywords:** regenerated cellulose, viscose rayon, biobased polymers, fique, agro-industrial wastes, cellulose pulp

## Abstract

Fique fibers, native to Colombia, are traditionally used for ropes and bags. In the extraction of long fibers for these purposes, the same amount of short fibers is generated; the short fibers are then discarded in the soil or in landfills. This agro-industrial waste is cellulose-rich and can be potentially developed into new biobased products. As an alternative use for these fibers, viscose regenerated fibers with potential applications in the textile industry were developed. Fique waste fibers were pulped (to produce fique cellulose pulp, FCP) using a 3^3^ design of experiment (DOE) to adjust the variables of the whitening treatment, and DOE analysis showed that time and hydrogen peroxide concentration do not have a significant effect on non-cellulosic remotion, unlike temperature. The behavior of this pulp in the production of viscose was compared against that of commercially available wood cellulose pulp (WCP). FCP showed a suitable cellulose content with a high degree of polymerization, which makes it a viable pulp for producing discontinuous viscose rayon filaments. Both pulps showed the same performance in the production of the viscose dope and the same chemical, thermal, and mechanical behavior after being regenerated.

## 1. Introduction

In recent years, chemical industries have been searching for environmentally friendly raw materials to use in their industrial processes. Several biobased polymers such as cellulose, starch, proteins, and oils have been successfully used in the formulation of many products, e.g., cellulose for rayon viscose fibers [1]. Cellulose is mainly isolated from wood (90–95 wt % of all pulp). However, this source has been questioned because it is related to environmental problems such as deforestation which impact on climate, hydrology, soils, and biodiversity [2].

In this context, non-wood sources with significant cellulose content such as cotton linters [3], waste bagasse [4], oil palm empty fruit bunches [5], and bamboo [6] have been investigated for the production of several industrial products such as viscose rayon textile fibers.

In Colombia, the livelihood of more than 70,000 peasant families depends on the production of 30,000 metric tons of fique natural fiber per year for the manufacture of ropes and sacks. However, in the extraction of long fibers for this purpose, the same amount of short fibers is generated and then discarded in the soil or in landfills. This agro-industrial waste is cellulose-rich, containing cellulose (63.0 wt %) as its main component, along with lignin (14.5 wt %), hemicellulose, and other minor components such as pectins and waxes [7]. Cellulose, which can be used for the manufacture of higher-value-added industrial products such as viscose rayon textile fibers, occurs at a higher proportion in this non-wood waste than in wood (38–52 wt %).

In the country of Colombia, more than 100 species of plants that produce natural fibers have been recognized [8]. One of these fibers is fique. Fique is considered a national fiber and grows in several agricultural conditions that include fertile lands and even dry or loose soils of tropical zones. These fibers have similar characteristics to other fibers such as jute and sisal [9]. The plants have stems that are 1.5 m tall; fleshy leaves that are 20 cm wide, arranged in the form of a green rosette; and white flowers [10].

Therefore, because fique short fiber presents a high-cellulose composition and is currently a waste product with no added value, in this work, cellulose pulp from fique short waste fiber was evaluated for viscose rayon production. A design of experiments was carried out to establish the conditions of time, hydrogen peroxide concentration, and temperature for fique cellulose isolation, and the best conditions were used to obtain the fique cellulose pulp (FCP) to be used in the manufacture of viscose rayon by a traditional method. Fique viscose rayon (FV) characteristics were evaluated and compared with those of viscose produced from wood cellulose pulp (WCP). FV exhibited similar chemical, mechanical, thermal, and morphologic properties to wood-cellulose-based viscose. To the best of our knowledge, viscose rayon manufacture from fique waste fiber cellulose has not been evaluated and reported previously.

## 2. Results and Discussion

### 2.1. Design of Experiment (DOE)

**Figure 1:**
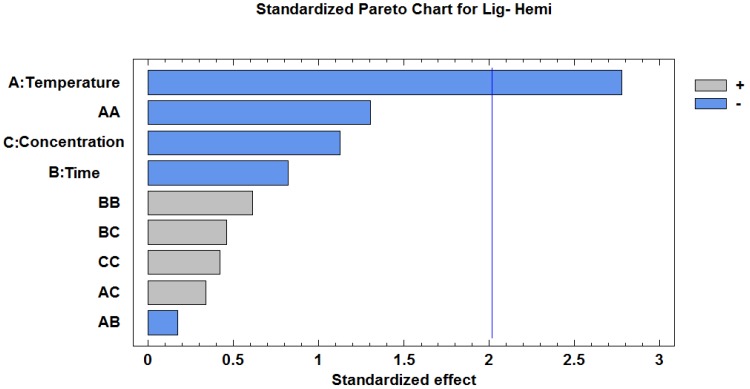
Pareto chart showing the main effect (A: temperature) that significantly contributes to non-cellulosic component removal, according to the results of the experimental design matrix.

As part of the experimental analysis, cellulose percentage data for each sample were analyzed using Statgraphics software. Table 1 displays the analysis of variance (ANOVA), which shows that time and concentration do not have a significant effect on the removal of non-cellulosic materials because their *p*-value is higher than 0.05, unlike temperature, which does have a significant effect on the removal of non-cellulosic materials. These results are exhibited in the Pareto diagram presented in Figure 1. The Pareto diagram shows that temperature is the only factor that has a significant effect on the reduction of non-cellulosic components. However, it must be considered that the Pareto principle is based on the fact that the observed variability is due to a few main effects. For an effect to be deemed as a main effect, it must exceed the standardized effect by two units *(x* axis) [11]. Therefore, we can deduce that the higher the temperature, the lower the content of non-cellulose components (this is valid for the temperature range evaluated in this study).

To verify the accuracy of this information, all assumptions were assessed, and we validated that all ANOVA assumptions had indeed been met. As concentration and time had no significant effects, in the present study, we proceeded to work with temperature, least time, and the lowest concentration of peroxide as the variables reported to save costs; therefore, the working conditions chosen were 80 °C, 30 min, and 1 wt %, respectively.

### 2.2. Physicochemical Characterization of Fique Cellulose Pulp (FCP) and Wood Cellulose Pulp (WCP)

**Figure 2:**
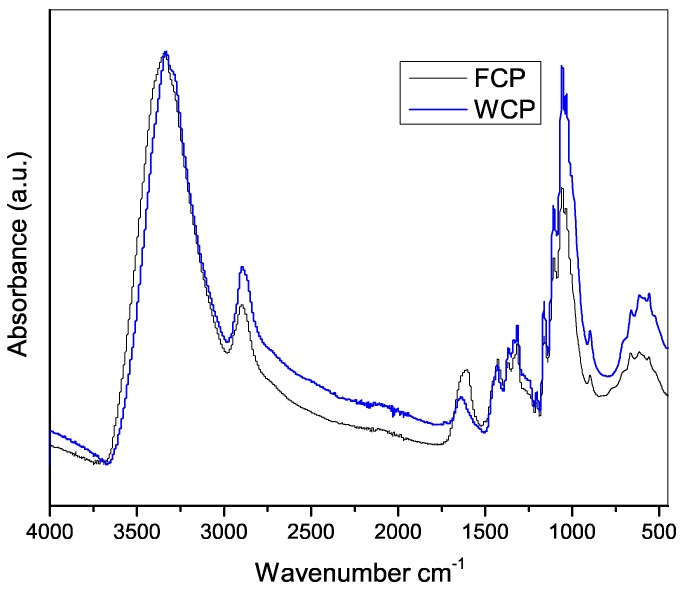
FTIR-ATR spectra of fique cellulose pulp (FCP) isolated at 80 °C, for 30 min, and with 1 wt % of peroxide (black line), and commercial wood cellulose pulp (WCP) (blue line).

FTIR spectroscopy was conducted to observe the chemical variations induced by the optimized treatment performed to obtain the FCP. This technique also evaluated WCP for comparative purposes. In both spectra (Figure 2), significant bands were presented at 3340, 2890, 1640, 1430, 1370, 1310, 1160, 1060, and 896 cm^−1^, which are consistent with the typical cellulose structure [12]. A band at 3340 cm^−1^ is typically caused by the stretching of the OH groups present in the structure [13], while the band at 2890 cm^−1^ corresponds to the stretching of the C–H bond. In both cases, the vibration at 1730 cm^−1^, typically caused by the acetyl and ester groups in the hemicellulose, pectins, and lignin, is absent, indicating that this non-cellulose component was removed during processing [13,14].

In addition, the spectra displayed typical bands for cellulose I, with strong bands at 1429 and 1111 cm^−1^ assigned to symmetrical bending of the CH_2_ and the stretching of the C–O bond, respectively, and a weak and wide band centered at approximately 897 cm^−1^, typical of glucose polymers linked by β-glucosidic bonds. Other bands at 1375 cm^−1^ (due to bending in C–H), 1335 cm^−1^ (due to bending of O–H in the plane), 1315 cm^−1^ (due to movement of CH_2_), 1277 cm^−1^ (due to bending of the C–H bond), and 1225 cm^−1^ (due to O–H in the plane) indicate the presence of crystalline regions within the structures.

The quantification of the main components cellulose, hemicellulose, and lignin using FTIR was performed for both the main virgin fique (FF) commodity and its cellulose pulp (FCP) used to produce viscose rayon; the results are presented in Table 2. FCP has approximately 19% more cellulose, 1% less lignin, and 8% less hemicellulose content compared with FF, which confirms the removal of non-cellulosic components. These results are in agreement with the reduction of the hemicellulose (2850 and 1730 cm^−1^ bands) and lignin (1500 and 1160 cm^−1^ bands) contribution bands evidenced by the infrared spectra.

Commercially available wood pulps generally have high cellulose values (>90 wt %) and a lower amount of lignin and hemicellulose as compared with FCP. The higher content of α-cellulose is ideal for the manufacture of continuous filaments of viscose rayon. However, FCP can be used for the production of cut filaments of viscose rayon, as suggested by Sixta et al. [16].

The intrinsic viscosity was measured to find the length of the polymer chains (degree of polymerization) present in both FCP and WCP. Table 3 displays the results obtained from the analysis of both samples. As these pulps originate from different sources, the chain length obtained from wood stem sources tends to be longer, owing to the supporting role it plays in its natural state, unlike the leaf cellulose in FCP. It should also be noted that an average degree of polymerization (DP) of 250–300 is sufficient for most viscose products [17]; therefore, the fique cellulose pulp meets and exceeds the minimum requirements for the polymerization degree.

Based on the foregoing and considering that FCP exhibits extremely similar characteristics to WCP, using FF for the formation of viscose rayon is, therefore, viable.

### 2.3. Characterization of the Fique Cellulose Viscose (FV) and Wood Cellulose Viscose (WV) Dopes

**Figure 3:**
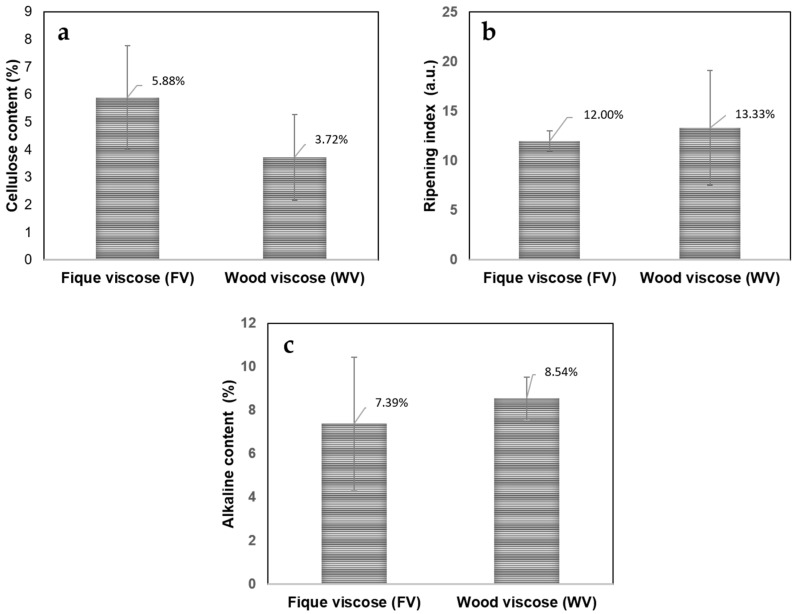
Cellulose content (**a**); ripeness index (**b**); and alkaline content (**c**) for both viscose dope solutions.

Figure 3 shows the values of the amount of cellulose, ripeness index, and alkaline content for both viscose dope solutions. The cellulose content present in FV is a few tenths higher than that present in WV; therefore, the difference between the cellulose contents in both samples is not statistically significant [18,19,20]. Those results are within the reported range in the literature (between 4 and 8), so the solutions can be considered suitable for the process of regeneration and solidification. Additionally, since the values of the ripening index are within the range of 11–14 mL, it can be concluded that the coagulation time is appropriate to obtain solid materials with an adequate percentage of crystallinity [21,22,23]. Finally, since a high amount of NaOH can affect the regeneration of the cellulose xanthate since it makes the exit of the CS_2_ from the solution difficult, the alkali content in the viscose solution should be between 4.6% and 8.0%, as is present in the FV dope. Therefore, with these value,s it can be said that the FV dope is suitable for the subsequent coagulation and solidification process, having a suitable viscosity for the extrusion (6000–4000 MPa) and a suitable ripening index (11–14 mL) to obtain a solution that coagulates in a reasonable time [24].

### 2.4. Characterization of Viscose Rayon Films Obtained from FCP and WCP

To validate whether the viscose rayon originating from the FV produced in this work has competitive characteristics as compared to viscose rayon produced from commercially available wood pulp (WV), FTIR, TGA, DSC, and mechanical tests of both viscose films were performed to compare their chemical, thermal, and mechanical features.

**Figure 4:**
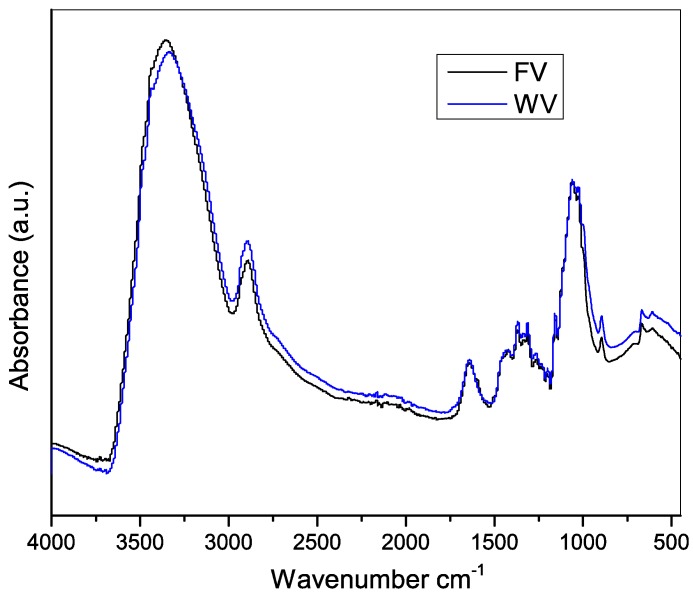
FTIR-ATR spectra of fique viscose (FV) (black line) and wood viscose (WV) (blue line) films.

FTIR testing of the viscose samples was performed to guarantee that the vibrations of the materials are consistent with the vibrations produced by the viscose rayon with predominant bands of cellulose II. Figure 4 shows the FTIR spectra of both the viscose rayon obtained from FV and that obtained from commercially available WV. Typical cellulose bands were observed at 3340, 2900, 1640, 1430, 1370, 1310, 1278, 1232, 1200, 1055, 1035, and 896 cm^−1^ in both spectra. The vibrations located at 3340 cm^−1^ and 1420 cm^−1^ can be attributed to the stretching of the OH groups and the vibrations of CH_2_ and O–C–H bonds, respectively, present in the cellulose II structure [25,26].

Furthermore, the absence of the peak at 1111 cm^–1^ is a characteristic present in this type of cellulose [12,27]. For cellulose I, the band corresponding to the β-glucosidic bonds is present at 897 cm^−1^; for the spectrum of the analyzed viscose, this peak is found at 891 cm^−1^, as is characteristic [28]. Based on these results, there is no notable difference between the absorptions of the two viscose products, and that, in both cases, the cellulose II structure predominates.

**Figure 5:**
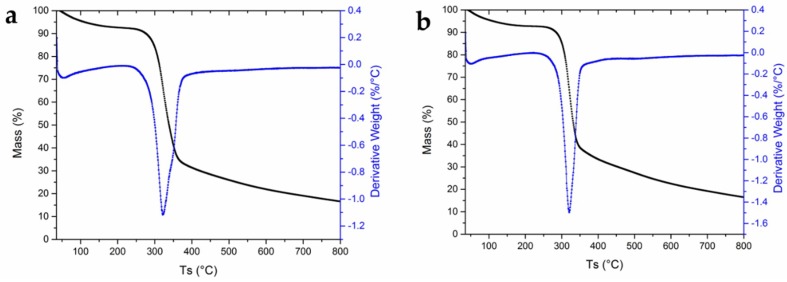
TGA curve (black line) and the corresponding derivative curve (blue line) of: fique viscose (FV) (**a**); and wood viscose (WV) (**b**) films.

In Figure 5a,b, thermograms for FV and WV, respectively, are shown, with the weight loss of the sample as a function of the temperature. For both cases, there is an initial loss of mass between 36.5 and 200 °C, with an approximate weight loss of 7%, which corresponds to the loss of water in the materials. The degradation of materials begins at around 263 °C, with a maximum at 321.405 °C and a mass loss of 37.04%, which ends at 393 °C. However, we can see that the peak for FV is much broader than that for WV. This is due to the presence of non-cellulose components with different degradation conditions, which was corroborated by the results of the sugars and lignin analysis [29]. Finally, the residual mass in both samples is approximately 19%, similar to that reported for conventional viscose rayon in the literature (around 14%) [30,31].

**Figure 6:**
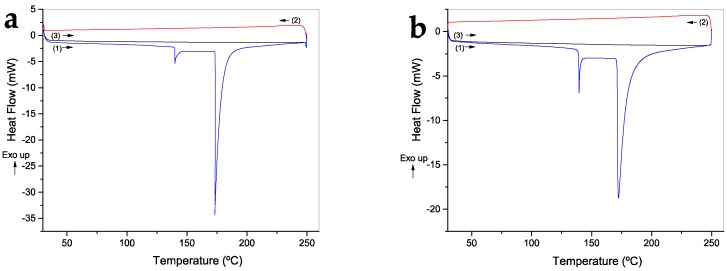
DSC curves of: fique viscose (FV) (**a**); and wood viscose (WV) films (**b**) ((1) first heating cycle, blue line; (2) cooling cycle, red line; and (3) second heating cycle, black line).

DSC was performed to determine the thermal behavior of the FV and WV samples (Figure 6). Both samples were heated up to 250 °C in a first heating (1), cooled (2), and heated again (3). In both cases, two endothermal events can be observed in the first heating at around 125 and 180 °C, which are related to the solvent evaporation caused by the presence of physically absorbed water in the structure, in which water molecules are restrained by hydroxyl groups in the amorphous region in cellulose II [27,32,33,34]. This solvent evaporation was also shown during the first loss of mass in the TGA curves and is corroborated by the absence of these events during the second heating in DSC curves; therefore, bound water was evaporated from the structure.

**Figure 7:**
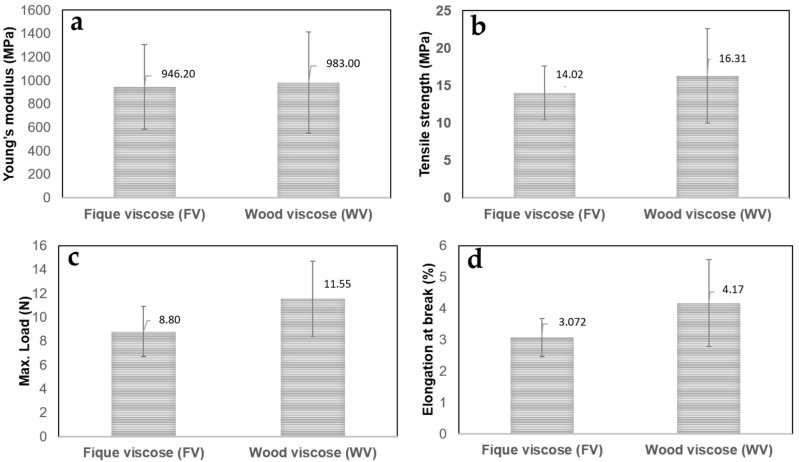
Mechanical behavior of fique viscose (FV) and wood viscose (WV) films: Young’s modulus (**a**); tensile strength at break (**b**); maximum load (**c**); and elongation at break (**d**).

Figure 7 displays the mechanical properties for FV and WV with their corresponding deviations. Here, it can be observed that there are no statistically significant differences between the properties of both viscose rayons; therefore, in terms of mechanical properties, both materials are extremely similar. The Young’s moduli of the films were in the 946–983 MPa range, the ultimate tensile strength varied between 14 and 16 MPa, the maximum load on average was 10 N, and the elongation at break varied between 3% and 4.7%.

**Figure 8:**
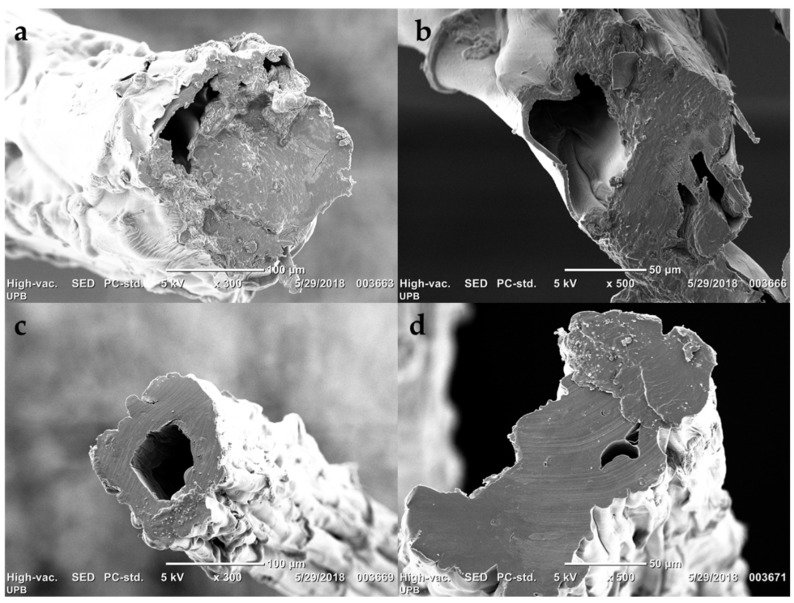
SEM of the cross section of: fique viscose fibers ×300 (**a**); fique viscose fibers ×500 (**b**); wood viscose fibers ×300 (**c**); and wood viscose fibers ×500 (**d**).

To validate the FV dope for filament manufacture, the dope was extruded and solidified; SEM images of the filaments are presented in Figure 8. All images exhibit a rough surface with a granular topography that is typical of viscose rayon morphology [31]. Filaments presented a wrinkled surface owing to the coagulation of the material [35]. In both cases, we can see that the cross section presents cavities formed generally by the solidification of the viscose [36].

On the other hand, the cross section of the fibers presents a lobed or circular serrated (jagged) shape with a highly rough/striated surface; this is characteristic of the coagulation process of viscose rayon fibers because this is found first on the surface and delays coagulation within the fiber [35,37,38].

## 3. Materials and Methods

### 3.1. Materials

Short fique fibers were kindly provided by *Asociación de Fiqueros y Artesanos de la cabuya del municipio de San Vicente Ferrer* (AFAS), Antioquia, Colombia. The following chemical agents were used: sodium hydroxide (Merck, Darmstadt, Germany), sodium hypochlorite (Alfa aesar, Tewksbury, MA, USA), carbon disulfide (Panreac, Darmstadt, Germany), sulfuric acid (Merck), zinc sulfate (Merck), and sodium sulfate (Merck). All chemical reagents were used with no further purification. Commercial wood cellulose pulp from pine was supplied by Colorquímica.

### 3.2. Fique Cellulose Isolation (FCP)

Fique short fiber was milled using a Retsch SM 100 cutting mill (Retsch GmbH, Haan, Germany) and sieved through a 40 mesh (ASTM). This fiber was treated with a 5 wt % NaOH solution (1:20) under mechanical stirring at 60 °C for 1 h and extensively washed with distilled water to neutral pH. A randomized design of experiment (DOE) for the determination of optimum bleaching conditions was carried out. Based on the results of the preliminary experiments (unpublished results), three independent variables were selected, i.e., time, temperature, and hydrogen peroxide concentration, and a three-level, three-factor DOE was used, assuming the cellulose content as the dependent variable. Table 4 and Table 5 present the coding of the levels of independent variables, the design matrix used for optimization, and the data. Statistical analysis was carried out using a one-way ANOVA on Statgraphics^®^; the lines in the graphs represent statistically representative differences between the groups.

The treatment that resulted in the highest cellulose content was used to process the fique cellulose pulp (FCP) to be used for viscose rayon preparation. Wood cellulose pulp (WCP) was used for comparative purposes.

### 3.3. Preparation of Fique Viscose Dope (FV)

Twenty grams of FCP pulp were impregnated with 19 wt % NaOH solution (1:20) under mechanical stirring for 1 h at room temperature, then pressed until it was 3 times its initial weight and crushed to obtain alkali cellulose. The alkali cellulose was left to age for 24 h and then was churned with 7.5 mL of 99.5 wt % liquid CS_2_ for 2 h to obtain sodium cellulose xanthogenate. The resulting material was dissolved in 50 mL of 18 wt % NaOH solution, filtered, deaerated, and ripened for 24 h. Finally, the viscosity of the ripened solution was adjusted with 8 wt % NaOH solution to 6380 mPa·s. Wood cellulose viscose (WV) was also prepared following the same process.

### 3.4. FV Film and Filament Preparation

Films were prepared by casting of ripened FV solution on a glass plate. The plate was dipped into a bath consisting of 10.5 wt % H_2_SO_4_, 25.04 wt % Na_2_SO_4_, and 1.65 wt % ZnSO_4_ at 65 °C for 15 min, followed by extensive washing with distilled water. Finally, the films were oven-dried at 60 °C for 48 h and stored in moisture-controlled desiccators.

The filaments were formed when the viscose solution was extruded through a spinneret and dipped into a coagulation bath. Filaments were washed and dried under the same conditions mentioned for films.

### 3.5. Physicochemical Characterization of Fique Cellulose Pulp

Samples from the different treatments in the experimental design were hydrolyzed by adding approximately 300 mg of the material to 3 mL of 72 wt % sulfuric acid. The mixture was heated in a water bath at 30 °C for 1 h and then diluted to 4 wt % sulfuric acid concentration with 79 mL of distilled water. The diluted mixture was heated to 121 °C for 60 min in closed tubes, then the material was cooled and filtered through a porous glass number 4 filter. The solid residue on the filter was dried at 105 °C and weighed for the determination of Klason lignin, or acid-insoluble lignin [39]. The filtrate was diluted with water to 250 mL and measured at 203 nm to determine the amount of acid-soluble lignin using a Thermo Evolution 600 UV–Visible spectrophotometer (Thermo Fisher Scientific, Waltham, MA, USA). An analysis of glucose and cellobiose in the soluble fraction was performed via high-performance liquid chromatography (HPLC) using a Merck Hitachi instrument (Hitachi High-Tech, Tokyo, Japan) with an Aminex HPX-87H column at 45 °C, eluted at 0.6 mL/min with 5 mM H_2_SO_4_ by a refractive index detector.

The FCP with higher cellulose content was chemically characterized by infrared spectroscopy using a Nicolet 6700 FTIR spectrometer (Thermo Fisher scientific) equipped with a single-reflection ATR and a type IIA diamond crystal mounted in tungsten carbide. The diamond ATR had a sampling area of approximately 0.5 mm^2^, where a consistent reproducible pressure was applied to every sample. Infrared spectra were collected at 4 cm^−1^ resolution and 64 scans were carried out.

The DP was determined from the intrinsic viscosity (η) using the Mark–Houwink equation (Equation (1)) [40]. The intrinsic viscosity was measured according to the ASTM D 1795 standard with cupriethylenediamine as the solvent.

(1) $$\;\left[\eta \right]=2.28DP^{0.76}\;$$

### 3.6. Physicochemical Characterization of Fique and Wood Viscose Films

The solidification capacity of both viscose dope solutions was evaluated based on the amount of cellulose, ripeness index, and alkaline content.

A quantity of 1.5 g of the viscose jelly was pressed into each of two watch glasses and separated into a light film-like layer. The watch glasses were immersed in a bath of sulfuric acid until the films detached from the watch glasses. The films were washed with plenty of water, and excess moisture was removed using a paper towel; the films were then dried at 105 °C in a forced convection oven for 2.5 h [17]. The cellulose content was calculated according to Equation (2):

(2) $$\;Cellulose\;content\;\%=\frac{\left(weight\;of\;sample\;after\;drying\;\left(g\right)\right)*100}{weight\;of\;viscose\;\left(g\right)}\;.\;$$

The ripeness index is also known as the ripening index or Hottenroth number, and is calculated as the required volume (mL) of ammonium chloride solution at 10 wt % to coagulate 20 g of viscose jelly solution diluted in 30 mL of water. This indicates how quickly the viscose coagulates, which depends on its quality, i.e., the faster it coagulates, the lower the quality. The desired value is between 11 and 14 mL for better-quality viscose, especially for fiber production [20,21].

To determine the alkaline content, 2.5 g of the viscose jelly were weighed and dissolved in 100 mL of deionized water with constant agitation for 30 min. The solution was brought to boiling point, following which 10 mL of 0.5 η of sulfuric acid were added, and the solution was allowed to cool down. When the sample reached room temperature, it was titrated with 1 M NaOH using phenolphthalein, until a change in color was noticed. The alkali content was calculated according to Equation (3) [17]:

(3) $$\;Alkali\;content\;\%=\frac{\left(V_{1}*2*n_{1}\right)*\left(V_{2}*n_{2}*40\right)}{\left(m_{v}*1000\right)}*100\;$$

where *V*_1_ is the volume of H_2_SO_4_ (mL), *V*_2_ is the volume of NaOH (mL), *m_v_* is the mass viscose (g), *n*_1_ is the molar concentration of H_2_SO_4_ (mol/dm^3^), and *n*_2_ is the molar concentration of NaOH (mol/dm^3^).

Infrared spectroscopy analysis was carried out to analyze the chemical structure of the FV and WV films, following the conditions described above.

Mechanical characteristics were evaluated on 30 specimens per sample in an Instron 5582 universal machine (Instron, Norwood, OH, USA), according to the ASTM D 882 standard, using a load cell of 50 N at 10 mm/min and grip distance of 22 mm. All results were obtained with a standard deviation of less than 10%.

Thermogravimetric analysis (TGA, Mettler Toledo, TGA/SDTA85IE/ILF/1610) was performed to study the thermal degradation behavior of the composite samples. The TGA apparatus was flushed with a nitrogen atmosphere, and 10 mg samples was used. Each specimen was heated from room temperature to 800 °C at a rate of 10 °C/min. Differential scanning calorimetry (DSC, TA Q2000 series, TA instruments, New Castle, DE, USA) was used to acquire thermograms under N_2_ flow. Samples (5 mg) were placed in hermetically closed DSC crucibles and heated from 0 to 250 °C at 10 °C/min

Scanning electron microscopy (SEM, Jeol JSM 5910 LV operated at 10 kV, Jeol, Tokyo, Japan) was used to image the fracture surfaces of films deformed by tension and the extruded filaments of FV and WV. Before SEM analyses, all specimens were coated with gold/palladium using an ion sputter coater for 5 min.

## 4. Conclusions

In the present study, a cellulose isolation process was satisfactorily performed using fique and less-polluting treatments to obtain a pulp with cellulose content and polymerization grade (DP) appropriate for producing viscose rayon filaments. Likewise, the FV dope presented a suitable viscosity for the extrusion (6000–4000 MPa) and a suitable ripening index (11–14 mL) to obtain a solution that coagulates in a reasonable time in the regeneration and solidification process. After solidification, FV presented the same chemical cellulose II structure and the same mechanical behavior as did viscose obtained from wood cellulose pulp (WCP). However, its maximum degradation temperature and melting temperature were slightly higher due to the presence of traces of non-cellulose components containing aromatic rings in their structures; these units provide greater thermal stability to the material.

Finally, the fique viscose dope was successfully validated by the extrusion, coagulation, and soldering of filaments with extremely similar morphologies to those of commercial filaments. These results show that fique short fiber is a competitive potential raw material for producing viscose rayon. Given its characteristics, it may be used to produce cut filaments with applications for the textile industry.

Developments such as these seek to generate added value for agro-industrial waste products such as the fique short fiber discarded from the process of obtaining the long fiber only commercialized for rope and sack production; new uses for the short fiber have the potential to generate better income for thousands of peasant families in Colombia.

## Figures and Tables

**Table 1:** ANOVA for the content of non-cellulosic materials.

Source	Sum of Squares	Df	Mean Square	F-Ratio	*p*-Value
A: temperature	30.64	1	30.64	7.73	0.0082
B: time	2.68	1	2.68	0.68	0.4160
C: concentration	5.03	1	5.03	1.27	0.2666
AA	6.72	1	6.72	1.70	0.2002
AB	0.12	1	0.12	0.03	0.8637
AC	0.45	1	0.45	0.11	0.7383
BB	1.49	1	1.49	0.38	0.5433
BC	0.84	1	0.83	0.21	0.6495
CC	0.69	1	0.69	0.18	0.6777
lack-of-fit	35.97	17	2.11	0.53	0.9187
pure error	162.57	41	3.96		
total (corr.)	247.90	67			

**Table 2:** Analysis of virgin fique (FF), fique pulp (FCP), and wood pulp (WCP) components.

Components	Virgin Fique (FF)	Fique Pulp (FCP)	Wood Pulp (WCP) [15]
Cellulose (wt %)	50.5	69.1	96.8
Lignin (wt %)	13.9	12.8	0.1
Hemicellulose (wt %)	14.6	6.6	3.1
Others (wt %)	21	11.5	-
Total	100	100	-

**Table 3:** Intrinsic viscosity (η) and degree of polymerization (DP) of FCP and WCP.

Sample	Intrinsic Viscosity (dg/L)	DP	Chain Length (nm)
Fique cellulose pulp	6.77	1286.94	662.77
Wood cellulose pulp	8.96	1703.64	877.37

**Table 4:** Levels of the factors in the employed experimental design.

Independent Variables	Code	Levels		
		−1	0	1
Temperature (°C)	A	60	70	80
Time (min)	B	30	45	60
Peroxide concentration (%)	C	1	3	5

**Table 5:** Matrix standard with three-level factorial design with cellulose content as the response variable and non-cellulose (hemicellulose and lignin) content.

Factors				Cellulose (%)	Hemicellulose (%)	Lignin (%)	Others (%)
Treatment No.	A	B	C				
1	70	30	5	63.97	12.86	9.90	13.27
2	60	30	3	61.10	11.70	10.95	16.25
3	70	30	3	65.17	12.43	10.10	12.30
4	80	30	5	61.90	11.86	10.16	16.08
5	80	60	3	62.53	12.23	9.13	16.11
6	80	60	5	61.10	12.15	9.75	17.00
7	60	45	5	60.67	11.86	11.26	16.21
8	70	30	1	60.67	12.26	12.10	14.97
9	70	60	5	61.06	12.50	10.36	16.08
10	80	30	3	59.03	11.83	11.43	17.71
11	80	45	5	66.00	11.66	8.23	14.11
12	60	30	5	63.43	10.83	12.43	13.31
13	60	60	5	63.43	12.30	11.40	12.87
14	80	45	3	64.60	12.46	9.86	13.08
15	70	60	3	64.65	11.30	9.80	14.24
16	70	45	1	58.10	12.50	11.05	18.35
17	60	30	1	62.80	12.53	12.16	12.51
18	60	45	3	62.63	12.33	10.30	14.74
19	80	60	1	62.90	12.50	8.76	15.84
20	70	45	3	63.75	13.10	10.55	12.60
21	70	45	5	61.27	12.70	9.33	16.70
22	70	60	1	60.93	14.83	9.70	14.54
23	80	30	1	63.10	12.10	10.00	14.80
24	60	60	1	60.50	12.50	10.25	16.75
25	60	45	1	70.00	13.00	12	5.00
26	80	45	1	60.15	12.15	8.60	19.10
27	60	60	3	59.90	12.66	9.96	17.48
